# Combined methylation mapping of 5mC and 5hmC during early embryonic stages in bovine

**DOI:** 10.1186/1471-2164-14-406

**Published:** 2013-06-18

**Authors:** Béatrice de Montera, Eric Fournier, Habib Allah Shojaei Saadi, Dominic Gagné, Isabelle Laflamme, Patrick Blondin, Marc-André Sirard, Claude Robert

**Affiliations:** 1 Centre de Recherche en Biologie de la Reproduction, Institut des Nutraceutiques et des Aliments Fonctionnels, Université Laval, Québec, QC, G1V 0A6, Canada; 2 L’Alliance Boviteq Inc., Saint Hyacinthe, QC, J2T 5H1, Canada

**Keywords:** Embryo, Methylation, Hydroxymethylation, Differentiation, *In vitro* conditions

## Abstract

**Background:**

It was recently established that changes in methylation during development are dynamic and involve both methylation and demethylation processes. Yet, which genomic sites are changing and what are the contributions of methylation (5mC) and hydroxymethylation (5hmC) to this epigenetic remodeling is still unknown. When studying early development, options for methylation profiling are limited by the unavailability of sufficient DNA material from these scarce samples and limitations are aggravated in non-model species due to the lack of technological platforms. We therefore sought to obtain a representation of differentially 5mC or 5hmC loci during bovine early embryo stages through the use of three complementary methods, based on selective methyl-sensitive restriction and enrichment by ligation-mediated PCR or on subtractive hybridization. Using these strategies, libraries of putative methylation and hydroxymethylated sites were generated from Day-7 and Day-12 bovine embryos.

**Results:**

Over 1.2 million sequencing reads were analyzed, resulting in 151,501 contigs, of which 69,136 were uniquely positioned on the genome. A total of 101,461 putative methylated sites were identified. The output of the three methods differed in genomic coverage as well as in the nature of the identified sites. The classical MspI/HpaII combination of restriction enzymes targeted CpG islands whereas the other methods covered 5mC and 5hmC sites outside of these regions. Data analysis suggests a transition of these methylation marks between Day-7 and Day-12 embryos in specific classes of repeat-containing elements.

**Conclusions:**

Our combined strategy offers a genomic map of the distribution of cytosine methylation/hydroxymethylation during early bovine embryo development. These results support the hypothesis of a regulatory phase of hypomethylation in repeat sequences during early embryogenesis.

## Background

Epigenetic marks are defined as enzyme-mediated chemical modifications of DNA and/or of its associated chromatin proteins. Being epigenetic, these marks do not alter the primary sequence of DNA, but nevertheless contain information that may be heritable in daughter cells or potentially transmitted to downstream generations through germ cells. In plant and mammalian DNA, the organic base 5-methylcytosine occurs at CpG sites and accounts for 1–6% of nucleotides. Cytosine methylation being the most stable epigenetic mark, it is involved in three vital biological processes in mammals: embryogenesis, genomic imprinting and the regulation of transcription [1-3]. Methyl cytosine also occurs at CHG and CHH sites (where H = C, T, or A) especially in stem cells [3,4]. Another modified organic base, 5-hydroxymethylcytosine (5hmC), whose presence was revealed in the nervous system forty years ago [5], has been recently detected in mice, rats, rabbits and cattle [6-9]. The enzyme Tet1 has been shown to catalyse the conversion of 5mC to 5hmC [7] and is reported to regulate developmental processes, neurogenesis and cellular differentiation [10]. Oxidative damage to methyl-CpG sequences has been reported to inhibit the methyl-CpG binding domain of methyl-CpG binding protein 2 [11] and 5hmC has now been identified as the cause of so-called “oxidative demethylation” [12,13]. The Tet1 catalytic domain is capable of hydroxylating 5mC *in vitro* at CHG and CHH sites [14]. The closely related enzymes Tet2 and Tet3 appear to have compensatory roles in undifferentiated tissues or redundant tissue-specific roles in differentiated tissues [15] and have been shown *in vitro* to play an important role in mouse ES cell lineage specification. Their depletion also has an impact on pluripotency-related genes [16].

These recent data raise questions about the role of 5hmC in methylation reprogramming processes inside the mammalian zygote. DNA methylation is currently associated with loss of pluripotency and differentiation, even though DNA methylation levels do not change very much during differentiation in mice ES cells [17]. Moreover, genes associated with pluripotency and germ-line specific genes actually gain methyl groups in the zygote [18,19]. This indicates that the methylation process during tissue-differentiation is not general and that gene-specific or lineage-specific demethylation can occur through the action of Tet1 [16,17,20]. Several questions remain, amongst which two are of primary interest: which genomic sites are actually modified during embryo development, and what are the contributions of 5mC and 5hmC to these changes.

Embryo methylation profiles have been reportedly altered through *in vitro* procedures [21]. Demethylation has been reportedly delayed in IVF-derived and ICSI-derived rat embryos in comparison to *in vivo* fertilization [22]. The simultaneous study of both 5mC and 5hmC thus could be of great value for the comparison of *in vitro* and *in vivo* embryos. Given that bovine developmental timing closely follows human developmental timing, that the impact of *in vitro* procedures on the bovine methylome has been documented [23,24] and that a fully annotated *Bos taurus* genome is available, cattle offer a good model for the study of methylome and hydroxymethylome changes during early development in response to *in vitro* constraints.

Oxidative Bisulfite sequencing (oxBS-Seq), a recently developed method for quantifying 5mC and 5hmC, uses selective oxidation of 5hmC to 5-formylcytosine (5fC) to enable bisulfite conversion of 5fC to uracil. As 5mC are the only cytosines not converted to uracil by the procedure, sequencing a sample treated with oxBS allows their identification. By combining these results with those of traditional bisulfite sequencing (BS-Seq), which leaves both 5mC and 5hmC unchanged, the levels of both epigenetic marks can be assessed through comparative data analysis [25]. However, such sequencing-heavy approaches, where each sample must be processed and then sequenced at least twice, are not compatible with the generation of multiple profiles from multiple samples in a cost-effective manner [3]. Alternative high-throughput strategies focus on reducing sample complexity by using methylation profiling, either through genomic DNA restriction by methyl-sensitive endonucleases, bisulfite conversion strategies or immuno-precipitation using methyl-binding proteins or antibodies against methylcytosine-containing sites [26,27]. Some promising new methods include GLIB (Glucosylation, periodate oxidation, biotinylation) [14,28] and the anti-CMS protocol (bisulfite conversion of 5hmC to cytosine-5-methylenesulphonate (CMS) followed by immunoprecipitation using an antibody against CMS) [29]. However, using the current protocols associated with these methods, we cannot meet the specific constraint of embryonic epigenome analysis, which entails being able to work with a reduced amount of genomic DNA (< 200 ng) corresponding to a minimal amount of pooled embryos.

We speculate that a well-chosen combination of both methylation profiling and sequencing-based approaches should help meet the challenge of building the methylome and hydroxymethylome profile of early mammalian embryos. Since methylation patterns are tissue-specific, information on the genome-wide distribution of 5hmC is still scant for human embryos and model species like *Bos taurus*. Consequently, in order to perform a first exploration of 5mC and 5hmC representation in mammalian embryos, we sought to develop a strategy for the rapid generation of differently methylated regions. The methyl-sensitive restriction-based strategy proposed herein is a high-throughput approach providing a genomic mapping of 5mC and 5hmC-containing sequences with uniform coverage by using different sets of methyl-sensitive enzymes. We combined three protocols to obtain a comprehensive survey of candidate 5mC and 5hmC sites during early bovine development: an adapted methyl-sensitive representational difference analysis (Me-RDA) protocol [30] using a MspI/HpaII isoschizomer system [31], a variation using FspBI/BfaI isoschizomers to reveal hydroxymethylation (HMe-RDA) and enzymatic cocktail-enrichment ligation-mediated PCR protocol (“HELP cocktail”) adapted from Schumacher et al. (2006) [32]. This combined strategy allowed the profiling and mapping of an important amount of methylated and hydroxymethylated sites in early developmental stages.

## Results

All three of the library construction methods are based on selective restriction cleavage using some combination of methylation/hydroxymethylation-sensitive and methylation/hydroxymethylation-insensitive enzymes. The aim of each restriction enzyme combination was to identify both 5mC and 5hmC as well as to offer extended genomic coverage.

### Me-RDA strategy: targeting 5mC sites in CpG islands

Methylation-sensitive representational difference analysis (Me-RDA) is a protocol composed of traditional RDA methodology [33] which combines methyl-sensitive and methyl-insensitive endonucleases for the identification of hypo- and hyper-methylated DNA fragments [34,35]. Its underlying mechanism uses PCR, subtractive hybridization and kinetic enrichment to amplify a set of restriction fragments present in one sample (Tester, cleaved with a methyl-insensitive endonuclease) but not the other (Driver, cleaved with a methyl-sensitive endonuclease). Adaptors are ligated only to the Tester fraction. When subtractive amplification is performed, only Tester-Tester homodimers are exponentially amplified while Tester-Driver heterodimers follow a linear amplification and progressively become underrepresented. The resulting enrichment in Tester-Tester homodimers constitutes indirect evidence that there are no corresponding amplicons in the Driver representation, indicating that the corresponding restriction sites remained uncut, presumably because they were completely or partially methylated.

**Figure 1:**
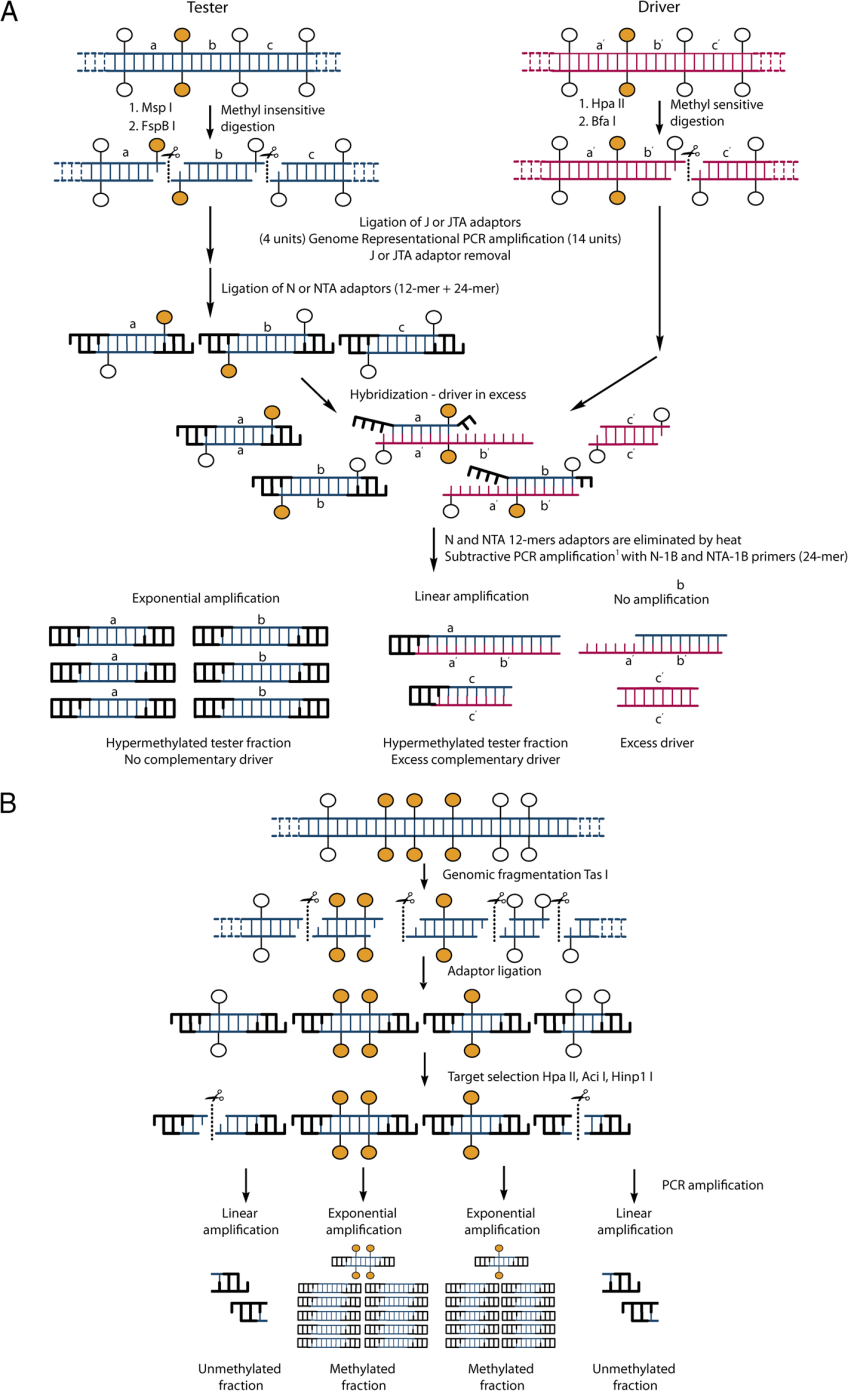
**Schematic illustration of procedures for the three methodologies used to survey embryo methylome and hydroxymethylome. A**) Me-RDA and HMe-RDA methodologies use hybridization and subtraction between Tester DNA cleaved with an insensitive enzyme and Driver DNA cleaved with a sensitive isoschizomer. White circle: unmethylated cytosine; orange circle: methylated cytosine. ^1^ The subtractive PCR includes a single-strand DNA digestion step by a mung bean nuclease after 10 cycles of amplification and is followed by 20 cycles of amplification. **B**) HELP Cocktail methodology. Following TasI ligation mediated-PCR, amplification products are digested with a methyl sensitive enzymatic cocktail. Methylated fragments remain uncut, and are subject to exponential amplification in a final PCR.

**Figure 2:**
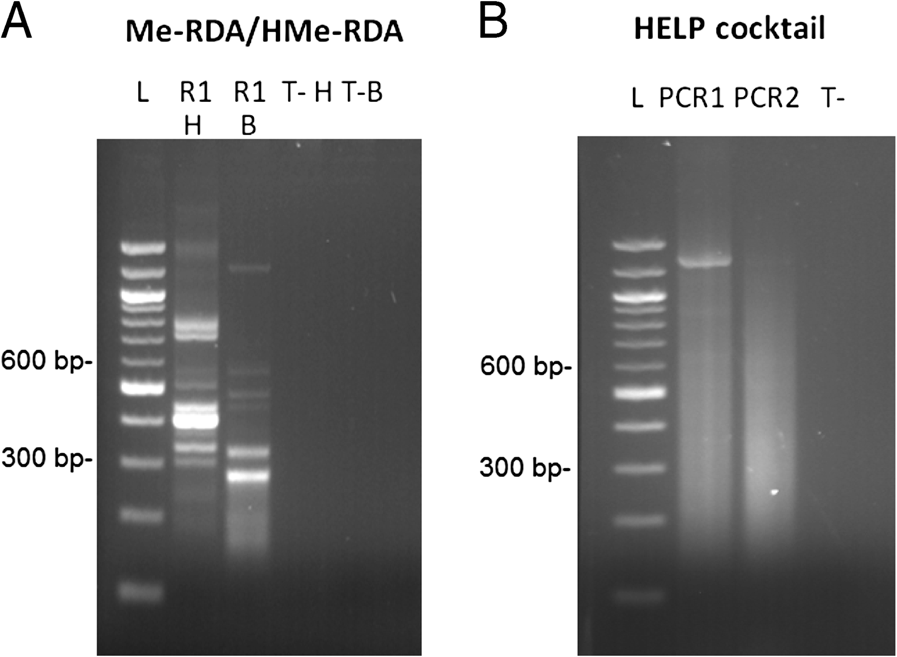
**Resulting smears of amplifications of blastocyst DNA from the three protocols. A**) Resulting smears of amplifications from Me-RDA and HMe-RDA methodologies. One round (R1) of kinetic enrichment results in a HpaII site-enriched smear (H) and a BfaI site-enriched smear (B) with PCR control for HpaII (T-H) and PCR control for BfaI (T-B). L: 100 pb ladder. **B**) Resulting smears from HELP Cocktail methodology. TasI ligation mediated-PCR (PCR1). Final PCR (PCR2).

Our strategy for the enrichment of methylated genomic loci is based on a modified protocol of Me-RDA and is illustrated in Figure 1A. It uses MspI as its methyl-insensitive endonuclease, and its isoschizomer HpaII as its methyl-sensitive endonuclease [31]. As the cleaving activity of both MspI and HpaII have been shown to be either inexistent or severely inhibited in the presence of an internal 5hmC [36], only 5mC bearing sites should be enriched by the procedure. Enrichment of the MspI fraction is confirmed when intense bands are revealed by electrophoresis. Bands for the MspI fraction could be obtained following a single round of subtractive amplification using a 1: 100 hybridization ratio of Tester and Driver DNA (Figure 2A, smear H). We chose hybridization conditions (>24 h) where the cot value allows nearly 100% of complementary template hybridization in complex genomes, including all non-repetitive sequences. The subtraction having been successful, we chose to limit it to a single round in order to target partially methylated sites, which are underrepresented and could thus be eliminated through a second round of subtraction. The experimental range of validated amplicons for RDA methods is between 100 bp and 4 Kb, depending on the enzymes used [30,33]. In the present study, the validation range for MspI/HpaII Me-RDA amplicons was between 100 bp and 1 Kb (data not shown).

### HMe-RDA strategy: targeting 5hmC sites

Given the potential involvement of the recently discovered 5hmC mark in the epigenetic regulation of important biological processes, we decided to develop a modified Me-RDA protocol that uses a hydroxymethylation-sensitive endonuclease. By reviewing the restriction enzyme database REBASE [37], we found a pair of isoschizomers, namely BfaI (sensitive) and FspBI (insensitive) to assess the hydroxymethylation status of cytosines. Following the Me-RDA experimental design, Tester DNA was cleaved with FspBI and Driver DNA was cleaved with BfaI (see method, Figure 1A). By using the same method to obtain hydroxymethylome data as was used to obtain methylome data, it was expected that the datasets would be comparable. Furthermore, in order to target both methylation and hydroxymethylation (or other base modifications) in the same reaction, it is possible to use a cocktail containing several restriction enzymes providing –TA protruding ends. This can be achieved by extending the HMe-RDA protocol into an RDA cocktail protocol, using the same set of JTA and NTA primers. For simple, double or triple cleavage generating –TA ends, we designed adaptors creating a new BfaI/FspBI site for subsequent adaptor removal. As a preliminary test, we successfully performed a Me-RDA cocktail protocol with double digestion of two –TA overhang restriction enzymes (BfaI and Tru1I) (Figure 2B, smear B).

### HELP cocktail strategy: targeting 5mC in non-CpG regions

A study from Schumacher and colleagues describes the benefits and limitations of strategies targeting both unmethylated and hypermethylated fractions [32]. In spite of showing *in silico* that the unmethylated fraction derived from HpaII cleavage results in ~ 22 times more fragments than the hypermethylated fraction derived from MseI, they proposed an informative approach based on enrichment of the hypermethylated fraction, which can improve information on methylated sites in distinct genomic regions. We thus chose to complement our Me-RDA 5mC data with a protocol based on the Schumacher strategy, of which the hypermethylated DNA fragment enrichment relies on frequent cutter TasI (/AATT) cleavage (Figure 1B). TasI sites are infrequent within GC-rich regions, leaving most CpG islands intact. After ligation to AATT-overhang specific adaptors, the unmethylated and hypo-methylated fragments were cleaved with a cocktail of methyl-sensitive endonucleases (HpaII, Aci1I and HinP1I) in order to impede their further amplification. The resulting fraction was enriched in hypermethylated fragments that escaped digestion and was therefore subsequently amplified. On the resulting gel, we observed that the successful amplification of methyl-sensitive cocktail digestion transforms the electrophoretic smear by removing intense bands and reducing fragment size (Figure 2B: see smears PCR1 and PCR2, respectively before and after methyl-sensitive digestion).

### Deep sequencing data analysis

Using these three protocols, selected genomic libraries were prepared for blastocysts (Day-7) and elongated embryos (Day-12). The extent of DNA cleavage was assessed in terms of intact sites in methylation-insensitive digestions. Results show reactions are > 90% complete (Table 1). Read length averaged 235 bp for a total of 1,283,097 analyzed reads (Tables 1 and 2). The number of clean reads per library was between 71,459 and 281,619 (Table 2). The enzyme pair targeting 5hmC generated the lowest number of clean reads for both developmental stages (Table 1). The HELP cocktail strategy required an extra step to filter contaminating fragments that did not harbour an internal restriction site. In the HELP cocktail strategy, TasI cleavage generates fragments with protruding TasI ends that may or may not contain methyl-sensitive sites. Digestion by a cocktail of methyl-sensitive enzymes will cut TasI-TasI fragments if they contain unmethylated internal HpaII, AciI or HinP1I sites. Consequently, final HELP products should be enriched in TasI-TasI fragments containing methylated HpaII, AciI or HinP1I sites or fragments containing no such sites. *In silico* analysis was used to discard the latter fragments (without HpaII, AciI or HinP1P sites), which brought no information on genome methylation status. If redundancy is removed, the portion of HELP cocktail fragments containing at least one expected restriction site and considered as real positives is about 20% of the identified contigs (Table 1). The number of contigs was comparable for all libraries with an average of 31,039 ± 6,165 consensus sequences.

**Table 1:** Per-library metrics

	**Blastocyst embryos**			**Elongation embryos**		
**Methods**	**Me-RDA**	**HMe-RDA**	**HELP cocktail**	**Me-RDA**	**HMe-RDA**	**HELP cocktail**
	**Msp/Hpa**	**FspBI/BfaI**		**Msp/Hpa**	**FspBI/BfaI**	
Average raw read length (base pair)^a^	285 ± 121	235 ± 142	257 ± 113	274 ± 132	115 ± 87	241 ± 142
Reads with internal genomic digestion site^b^	17 429 (6.2%)	3547 (4.8%)	23 396 (10.0%)	15,245 (7.9%)	2134 (3.0%)	9037 (4.1%)
Putative methylated restriction sites within CpG Islands^c^	1198 (33.4%)	455 (2.1%)	313 (1.7%)	899 (34.8%)	546 (3.5%)	1583 (4.0%)

^a^With standard deviation. ^b^Number of cleaned reads (percentage of all cleaned reads) containing an intact site for a methyl-insensitive restriction endonuclease more than 10 nucleotides from the ends. Such sites are indicative of incomplete genomic cleavage. ^c^The percentage of putative methylated restriction sites within CpG islands over the total number of sites identified for this library. As a reference, CpG islands cover 1.9% of the bovine genome.

**Table 2:** Bioinformatics analysis pipeline for the identification of putatively methylated sites

	**Blastocyst embryos**			**Elongation embryos**			**All**
**Method**	**Me-RDA**	**HMe-RDA**	**HELP cocktail**	**Me-RDA**	**HMe-RDA**	**HELP cocktail**	
	**Msp/Hpa**	**FspBI/BfaI**		**Msp/Hpa**	**FspBI/BfaI**		
Raw reads from library	313 330	179 105	242 864	208 963	94 961	243 874	1 283 097
Cleaned reads^a^	281 619	72 855	234 149	192 163	71 459	218 874	1 071 119
Validated reads^b^	N/A	N/A	46 835	N/A	N/A	50 462	N/A
Consensus sequences^c^	33 123	29 416	31 604	25 616	24 963	41 784	186 506
Sequences with genomic alignments^d^	28 941	25 360	25 519	18 944	14 978	37 759	151 501
Sequences with unique alignment^e^	1890	11 772	14 712	1317	8858	30 587	69 136
Putative methylated restriction sites^f^	3634	21 352	18 810	2584	15 714	39 367	101 461

^a^Number of reads left after cleaning with SeqClean. Reads smaller than 25 nt after adapter trimming were discarded. ^b^For HELP, number of reads with an internal HpaII, AciI or HinP1I restriction site. ^c^Number of consensus sequences after clustering with a 97% identity threshold. ^d^Number of consensus sequences with a BLAT genomic alignment with 92% identity over 92% of the length of the transcript. ^e^Number of consensus sequences for which a single alignment fits the above criteria. ^f^Number of potentially methylated sites identified in the genome through the alignment and extension of reads. N/A = not applicable.

As expected, the genomic alignment of contigs resulted in a high proportion of mapped loci. For bovine genome sequences that could not be mapped, further analysis ruled out linker concatemerization. However the presence of numerous shorter contigs could not rule out potential chimeric fragments or the identification of still unmapped bovine genomic sequences. The proportions of unmapped contigs were the highest for libraries targeting 5mC in CpG islands and 5hmC in Day-12 embryos (Table 1). BLAT results also showed that a large proportion of contigs aligned with multiple locations of the genome. This situation was particularly evident for the method targeting 5mC in CpG islands (Me-RDA), since only 6-7% of the contigs could be positioned unambiguously on the genome. These proportions were much higher for the other methods with 46% (Day-7) and 59% (Day-12) for 5hmC contigs and 58% (Day-7) and 81% (Day-12) for 5mC contigs (Table 1). These values corroborated our results with respect to repetitive content as well as the positioning of the contigs within CpG islands. More than 30% of the contigs isolated using Me-RDA mapped to CpG islands whereas only 2-4% of the contigs found with the other methods mapped to these CpG-rich domains (Table 2).

### Chromosomal mapping of consensus sequences

In order to evaluate the qualitative distribution of mapped sequences along bovine chromosomes, a chromosomal mapping of the 69,136 putative methylated regions was generated and plotted alongside genomic coverage data for genes and CpG islands as well as a cytoband mapping adapted from Liao et al. [38]. An illustration of the mapping of all chromosomes can be found in Additional file 1: Figure S1, while the data itself can be visualized directly on the EmbryoGENE genome browser (http://www.emb-bioinfo.fsaa.ulaval.ca/). We observe that the combined site distribution follows neither CpG islands nor genes and covers the entire genome. Overall, all three methods provided a distribution across the entire genome and were shown to target different loci, thus providing wide coverage.

### Establishment of 5mC and 5hmC marks within repeated elements

Characterization of the repetitive elements within the consensus sequences of the libraries (Table 3) showed that Me-RDA sequences were enriched in satellite elements (87.7% and 91.7% of total bases for Day-7 and Day-12 embryos respectively). Furthermore, BTSAT4 accounted for 94.4% and 88.9% of satellite loci in the Day-7 and Day-12 embryo libraries respectively (Additional file 1: Table S2). Since centromeres and telomeres are repeat-rich regions, a genomic coverage plot of BTSAT4 elements was generated to confirm the increased presence of centromeric and telomeric loci within the contig collections generated by Me-RDA (Additional file 1: Figure S2). By comparison, the abundance of BTSAT4-associated loci in HMe-RDA libraries suggests a transition in the establishment of the 5hmC mark between Day-7 and Day-12, since this specific sequence went from accounting for 13.5% of repeat-containing contigs in the Day 7 library to 31.7% in the Day-12 libraries. In contrast, BTSAT4 showed low representation in the HELP cocktail libraries (0.9% and 0.5% for Day-7 and Day-12 respectively) (Additional file 1: Table S2).

**Table 3:** Repetitive content in consensus sequences

	**Blastocyst embryos**			**Elongation embryos**		
**Method**	**Me-RDA**	**HMe-RDA**	**HELP cocktail**	**Me-RDA**	**HMe-RDA**	**HELP cocktail**
	**Msp/Hpa**	**FspBI/BfaI**		**Msp/Hpa**	**FspBI/BfaI**	
Number of sequences with repetitive content^a^	29 777	19 996	27 055	21 490	11 058	26 453
	(89.9%)	(68.0%)	(85.6%)	(83.9%)	(44.3%)	(63.3%)
SINE^b^	0.6%	9.4%	47.2%	0.3%	6.4%	30.1%
LINE^b^	0.3%	30.9%	17.3%	0.1%	10.0%	10.5%
LTR^b^	4.6%	8.4%	2.1%	0.4%	5.4%	4.5%
Satellites^b^	87.7%	13.9%	3.9%	91.7%	25.6%	3.4%
Total^bc^	93.4%	63.3%	71.9%	92.6%	47.9%	50.0%

Repetitive content as identified by RepeatMasker. ^a^Number of sequences (percentage of total) within which repetitive content was found. ^b^Percentage of the total number of bases within the consensus sequences of the given library. ^c^As a reference, annotated repetitive elements cover 48.1% of the bovine genome.

Retrotransposons also occupy a large contingent of the identified repeated elements. Among the retrotransposons that were identified, the short interspersed elements were found more abundant in the HELP cocktail libraries, which were shown to target more 5mC outside of CpG islands than did the other two libraries. Moreover, these sequences seem to be more methylated in Day-7 compared to Day-12 embryos (Table 3). The long interspersed elements were proportionally more prevalent in the Day-7 than in the Day-12 libraries and this situation was more evident for the 5hmC marks (Table 3).

### Functional mapping between methods

**Figure 3:**
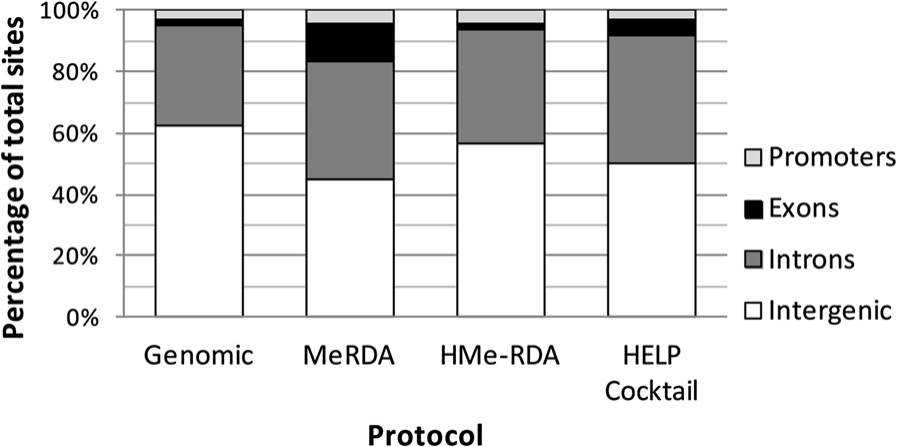
**Distribution of putative methylated/hydroxymethylated restriction sites among the genomic region types.** Genomic values are provided as a neutral reference.

To study the distribution of identified loci according to gene functional units, the genome was divided into promoter, exon, intron and inter-gene regions based on known gene structure. Gene promoter regions were defined arbitrarily as starting 5 kb upstream from the transcription initiation site. Since the coverage and distribution of putative methylated sites throughout the genome was similar across developmental stages, the dataset of both Day-7 and Day-12 embryos were combined for each library preparation methodology. Both epigenetic marks were found in every functional classification (Figure 3 and Table 4). The distributions of 5mC and 5hmC containing loci were quite similar across methods. The [Day-7 + Day-12] library proportions of promoter, exon, intron and inter-gene regions were found significantly different from the whole-genome proportions of those same regions (*P* < 10^-4^). Also, 5mC sites isolated using Me-RDA seemed more prevalent in exons.

**Table 4:** Distribution of types of genomic regions within which putative methylated/hydroxymethylated restriction sites were identified

**Region type**	**Genomic**^**a**^	**MeRDA**	**HMe-RDA**	**HELP cocktail**
Promoters	3.3%	4.1%	4.1%	3.0%
Exons	1.8%	12.5%	2.1%	5.0%
Introns	32.2%	38.6%	36.9%	42.1%
Intergenic	62.7%	44.7%	56.8%	49.9%

^a^All differences between genomic proportions and library proportions were found significant (*P* < 10^-4^).

### Mark-specific and stage-specific transition

**Figure 4:**
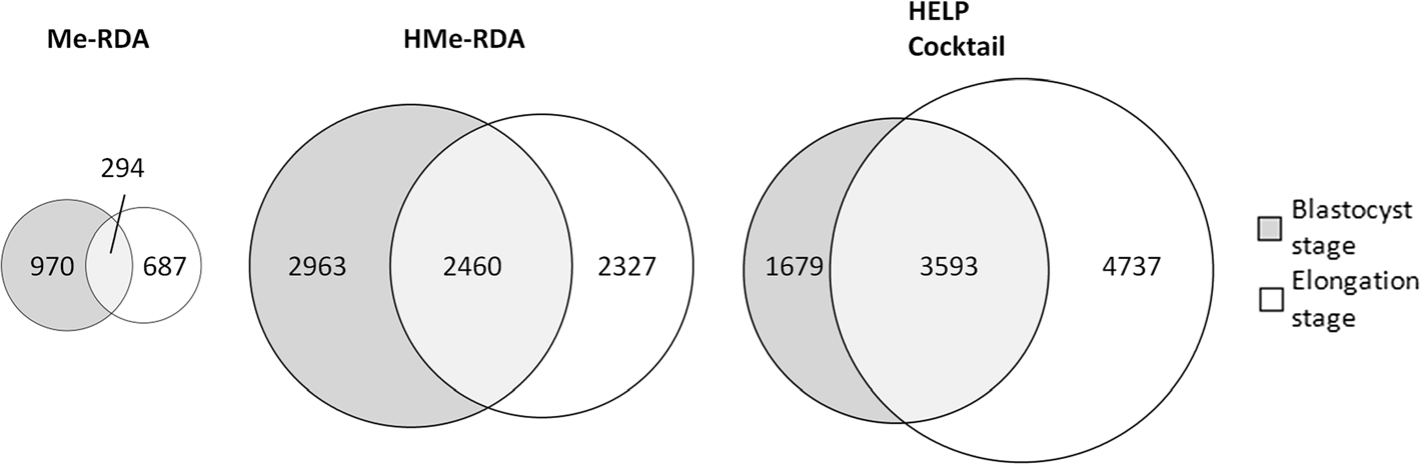
Number of methylated/hydroxymethylated restriction sites in the same genomic region for each protocol.

**Figure 5:**
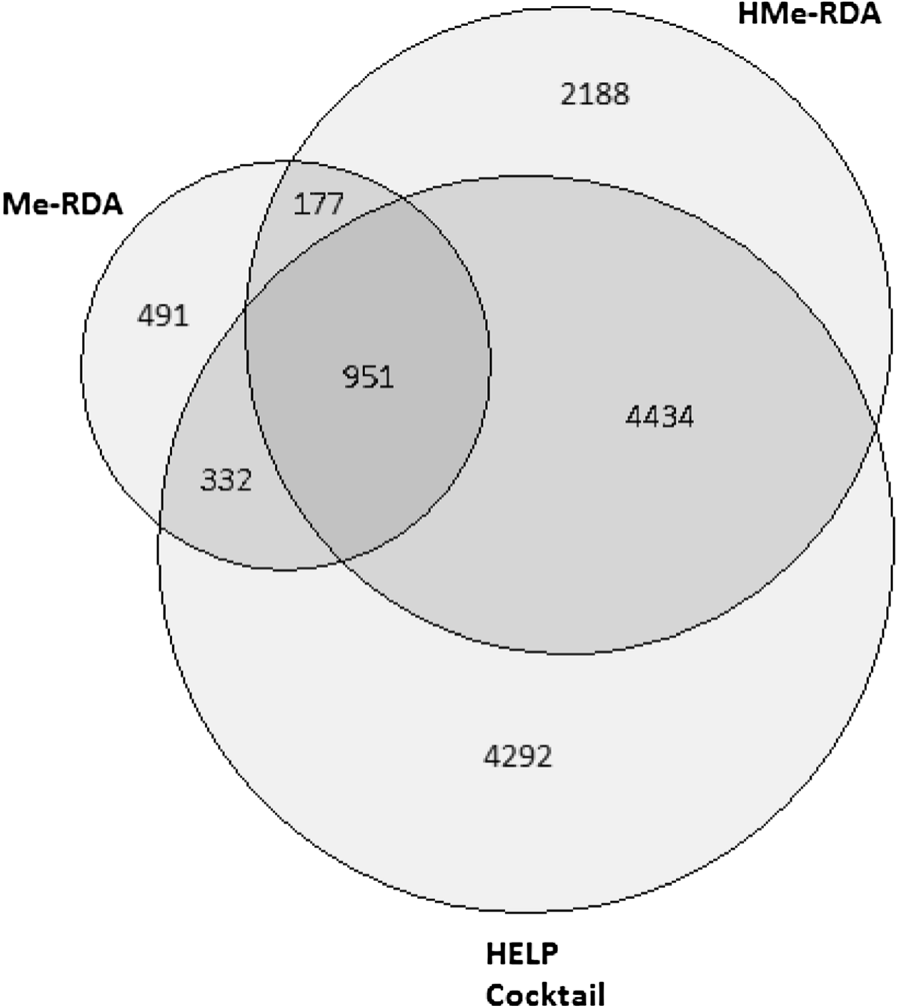
Overlap of methylated/hydroxymethylated genomic regions.

Since methylation is involved primarily in embryogenesis and development, while hydroxymethylation is involved notably in differentiation, methylated sequences may change during embryonic lineage differentiation taking place between the blastocyst stage (Day-7) and the beginning of the elongation stage (Day-12). To measure this, we compared the location of identified putative methylated sites between blastocyst and elongation stages for each method (Figure 4). The absolute number of Me-RDA 5mC regions is reduced from Day-7 to Day-12, but the elongated stage displays 687 specific new regions (70% of Day-12 regions). The number of HMe-RDA regions also decreased slightly from days 7 to 12, but Day-12-specific new 5hmC regions still represented 48.61% of total Day-12 regions. Conversely, the absolute number of HELP cocktail 5mC regions increased from Day-7 to Day-12 with the latter displaying 4,737 specific new regions (57% of Day-12 regions). Moreover, if we compare the portion of common or specific regions of all methods (Figure 5), the portion of specific Me-RDA 5mC regions is the lowest (25% of total Me-RDA regions) and is comparable to specific HMe-RDA regions portion (28% of total HMe-RDA regions), while the portion of specific HELP 5mC regions is noticeably higher (46% of total HELP cocktail regions). These observations show that the HELP cocktail method is likely an informative sensor of methylated transition sequences during elongation in bovine early embryos. The location of at least a quarter of 5hmC-containing sequences in different regions from 5mC illustrates a potential role of specific 5hmC regions during the blastocyst-to-elongation (Day-7 to Day-12) transition, which could be independent of 5mC location.

### q-PCR methylated and hydroxymethylated profiles

**Figure 6:**
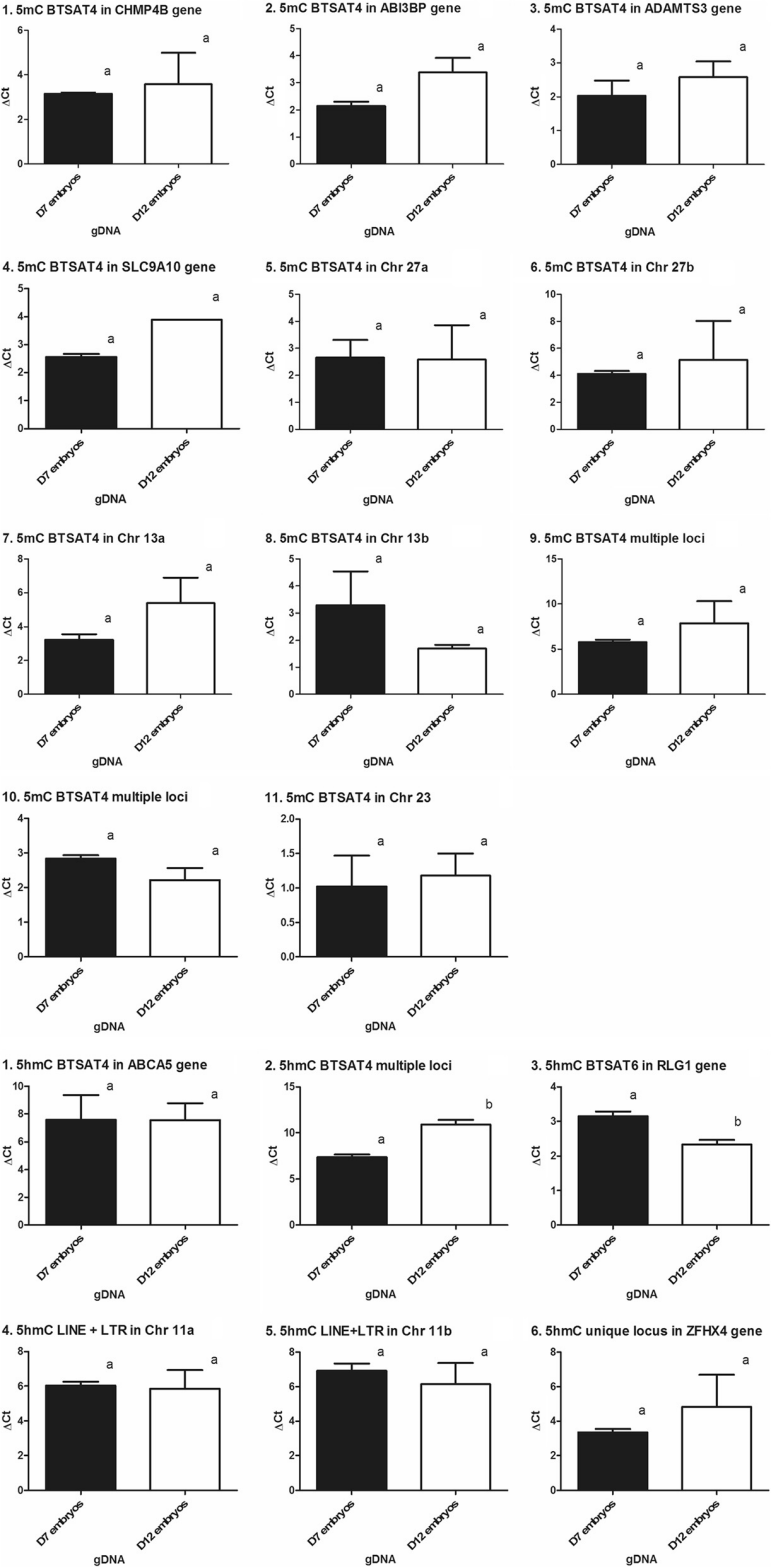
**Inter-stage comparison of methylation/hydroxymethylation status of selected subsets of loci.** Aliquots of samples were digested using either mark-sensitive or insensitive enzymes. The differences in amplification rates relates to the methylation (5mC or 5hmC) status. Different letters means statistically significant value *P* < 0.05*.*

During the initial validation tests performed by non-quantitative PCR on putative methylated candidates from granulosa cells obtained from one round of Me-RDA protocol, 78% of the 20 candidates showed differential intensity profiles between Tester and Driver (data not shown). For candidate embryonic sequences, subsets of selected repeated elements were targeted by q-PCR comparing the differences in amplification rates between aliquots of the same samples submitted to restriction digest either by mark-sensitive or insensitive enzymes [39] (Additional file 1: Table S1). For all three methods (Me-RDA, HMe-RDA, HELP Cocktail), 74% of the 23 sequences tested were validated to be partially methylated or hydroxymethylated. For both RDA based approaches combined (Me-RDA/HMe-RDA), 77% of candidate sequences were found to be methylated or hydroxymethylated and for HMe-RDA, all of the six tested candidates were found to be hydroxymethylated. On the 17 subsets of repeated elements selected from Me-RDA and HMe-RDA that validated, eleven and six candidates targeted methylation and hydroxymethylation respectively (Figure 6). None of the 5mC mark candidates showed significant differences between Day-7 and Day-12 stages. Amongst the six hydroxymethylation candidates, two loci containing satellite elements, BTSAT4 and BTSAT6, were tested and found significantly different between Day 7 and Day 12 embryos (*P* = 0.0035 and *P* = 0.0154, respectively). The 5hmC-containing BTSAT4 candidate displays multiple loci amongst the genome, whereas the BTSAT6 sequence includes the *RGL1* gene locus (5hmC Targets 2 and 3, Additional file 1: Table S1). The hydroxymethylation of this BTSAT6 sequence was significantly decreased in the *RLG1* locus of elongated embryos. The *RLG1* locus is reported to be involved in the cellular response to DNA damage and the maintenance of chromosomal integrity, both regulated by methylation [40,41].

## Discussion

This epigenomic survey based on methyl-sensitive restriction was inspired by earlier studies like the HELP assay [42,43] and unmethylated and methylated fraction enrichment strategies [32,44] but also by improvements in next-generation-sequencing-based technologies [26,45,46] and especially by the recently studied methylation mark 5hmC [15,47].

While adapting Me-RDA and HELP protocols to high-throughput objectives, the combined 5mC and 5hmC profiling strategy addressed methodological challenges and resulted in several advantages. Restriction-enzyme-based methods are usually used to enrich either the unmethylated or the hypermethylated fraction of gDNA. Since the HELP cocktail targets CpG-rich regions both inside and outside CpG islands with an estimated CpG dinucleotide coverage of 32% [32], it overcomes the main disadvantage of the simple HELP (simple HpaII digestion) assay which offers significantly lower genomic coverage [26]. In this combined approach, the HELP cocktail method, which provides enrichment of the hypermethylated fraction, is the most informative protocol. The HELP cocktail library displays the greatest coverage of gene and inter-gene regions, while the other protocols (Me-RDA and HMe-RDA) provided unique sets of complementary loci of interest, especially within satellite elements. The three protocols also display specific abilities. The TasI frequent cutter used in the HELP cocktail provides a fraction containing methyl-sensitive sites that escape GC site enrichment because of restriction site incompatibility. By including a subtractive amplification, Me-RDA and HMe-RDA can enrich rare fragments or sequences difficult to amplify because of fragment size or base content that would not have been enriched using a single ligation-mediated HELP PCR. Moreover, Me-RDA preferentially targets exon sequences, in contrast with HELP and HMe-RDA protocols, which can be explained by the large number of CpG islands in exons. Thus, the objective of discovering widely distributed candidate regions was achieved.

Differing reports on the genomic locations of 5hmC can be explained by either tissue or method-specific differences. The HMe-RDA dataset indicated high levels of 5hmC-containing sequences in inter-gene locations (57%). This was unexpected since the distribution of 5hmC in mouse Embryonic Stem cells (mES) shows 30% inter-gene location [10]. Nevertheless, a recent study on mouse pre-implantation embryos reported that highly methylated CpG islands are intra-gene, which can be explained by enhanced maintenance of methylation or resistance of non-imprinted sequences to demethylation [48]. Moreover, a microarray-based study of individual mouse blastocysts showed that 90% of the most methylated loci overlap with exons [49]. Since RDA strategies are known to amplify hypermethylated sequences, this can explain the overrepresentation of inter-gene locations and the high representation of repeats in the Me-RDA and HMe-RDA libraries.

A crucial advantage of applying PCR-based combined methylation and hydroxymethylation profiling to embryo epigenomics is the ability to reduce the requirement of input DNA by using optimized ligation and amplification. Each of the three protocols requires about 200 ng of genomic DNA, but improving PCR yield allowed successful representation starting with as little as 50 ng of DNA corresponding to an average of 200 pooled blastocysts. None of the current methylation or hydroxymethylation profiling methods (such as MethyCap-seq, HMedIP, GLIB or anti-CMS) can meet such constraints as they all require between 1 and 10 μg of starting DNA. Yet these approaches benefit from several advantages such as higher signal-to-noise ratio (especially for GLIB) [28] resulting in less false positives than in our restriction-based approach (Additional file 1: Table S3). Contrary to the random DNA fragmentation by sonication used within GLIB, our representation strategy is based on enzymatic restriction and yields predictable DNA fragments. This does not prevent our method from achieving uniform coverage of the bovine genome (Additional file 1: Figure S1), which met our objective of discovering and mapping new 5mC and 5hmC-containing sequences without any topological bias. In contrast with GLIB, our approach deliberately targets repetitive elements such as BTSAT4 and BTAST6 which are demonstrated by q-PCR validations to be candidates for transitory 5hmC marks during bovine embryogenesis.

Repetitive sequences are largely represented in a method-specific manner with prevalence of satellites in Me-RDA libraries and long interspersed elements (LINEs) in HELP libraries. Repetitive sequences are also represented in a stage-specific manner, notably for the HMe-RDA library. Previous studies using HpaII-methyl-sensitive RDA show the ability of the subtractive method to target repetitive sequences in tumors [50,51]. An interesting study in mice reports that 66% of isolated hypomethylated sequences correspond to LINE-1, LTR or SINE sequences [52]. The LINEs were proportionally more prevalent in the Day-7 than in the Day-12 HMe-RDA libraries confirming very recent data in mice showing that LINE-1 display an important amount of 5hmC and are reprogrammed during preimplantation development that is before differentiation processes [25]. In addition, it seems that satellite sequences in particular could be considered as possible candidates to target methylation and hydroxymethylation changes when comparing Day-7 to Day-12 bovine embryos. A previous comparison of methylation levels of repetitive loci during bovine development revealed that satellites I and II are hypo-methylated in blastocysts [53], consistently with studies hypothesizing that satellite demethylation occurs very early in the germ cell lineage, prior to entry into meiosis [54]. Given the hypothetical role of hydroxymethylation in oxidative demethylation, the increase in hydroxymethylation of bovine satellite sequences observed in HMe-RDA between Day-7 (13.9%) and Day-12 (25.2%) would be consistent with methylation removal associated with the differentiation of primordial germ cells in the early embryo genome which begins to happen in elongated bovine embryos [2]. Furthermore, it was shown that satellite I methylation decreased significantly in the trophectoderm but not in the embryo in somatic cell nuclear transfer embryos in comparison to *in vivo* embryos at the elongation stage (Day-12) [55]. The slight enrichment of 5hmC in satellite sequences in our blastocyst embryos and its associated putative demethylation could therefore be an effect of *in vitro* conditions.

One possible explanation for 5hmC enrichment of specific loci in Day-12 elongated embryos linked with putative demethylation could be the role of trophectoderm-specific methylation status. Indeed, Long Terminal Repeats (LTR) are significantly higher in HMe-RDA libraries, in particular at Day-12 stage, indicating a 5hmC enrichment within retrotransposons between blastocyst and elongation stages (Table 3). Consistent with this is a description of a role for endogenous retrovirus in trophoblast differentiation and placental development [56]. To address further on the question of a specific role of the trophectoderm in the overall 5hmC enrichment observed within LTR at elongation stage, embryonic tissue dissection will be necessary.

Another question of interest concerns the possibility of opposing 5mC and 5hmC marks. Several reports support the claim that 5hmC action maintains or promotes gene expression in opposition to 5mC [57] or represent a transition step and partially overcomes the silencing effect of 5mC [58]. Representation of certain 5mC marks and 5hmC marks was therefore expected to differ between Day-7 and Day-12 embryos.

The assumption regarding the specific regulatory role of 5hmC in differentiation processes is consistent with recent reports indicating that 5hmC is associated with specific sequence composition, poised chromatin configuration and gene expression upon differentiation [14,59,60]. In fact, activation of lineage-specific loci can occur either via a postulated 5mC demethylation pathway or through recruitment of transcriptional regulators that specifically recognize 5hmC and become activated in response to differentiation signals. For example, acquisition of 5hmC in cell-specific distal regulatory regions is expected to activate enhancers and participate in selective activation of tissue-specific genes in neural and adipose tissues [60]. A connection between 5hmC and regulatory elements appears likely in our data, since 5hmC is enriched upstream and downstream of Transcription Start Sites (TSS), while 5mC is enriched primarily downstream of TSS [14]. We observed that 4% of the resulting unique 5hmC sites for both the Day-7 and Day-12 embryos were located in the region 5 kb upstream from TSS indicating a possible regulatory role of 5hmC mark on its own.

More generally, our data raise questions about the role of 5hmC in the dynamic status of methylation inside the mammalian zygote during early developmental stages. An overall and differential loss of methylation happens in the first few days of development in mammals. In the paternal genome, demethylation occurs through an active enzymatic process, whereas the maternal genome apparently undergoes passive replication-dependant demethylation [61-63]. Recent data showed that 5hmC accumulated in the paternal pronucleus while 5mC decreased, supporting the model of Tet3 conversion of 5mC into 5hmC [9,64]. In mice, a second wave of demethylation in the maternal genome is normally completed by the time the embryo reaches the morula stage. Then, from the morula stage until the blastocyst stage, an increase in *de novo* methylation takes place [65]. But most recently, a study published by Smith and colleagues provides new insights into the two developmental transitions in DNA methylation occuring between the sperm and the zygote and between the early inner cell mass and the post-implantation embryo [66]. They observe that methylation levels reflect a global subtle but gradual decrease in mice post-implantation embryos. Consistently with our data, the most extreme changes are for sequences enriched for repeat elements: 18% of LINEs and 10% of LTR reduce significantly their methylation values. In our study, not only methylation levels at specific loci decrease, but also hydroxymethylation levels change indicating a role of 5hmC at specific repetitive sequences in the developmental transition around the period initiating foeto-maternal interactions. Furthermore, methylcytosines are likely to be targeted for complete demethylation since some 5hmC levels decrease between blastocyst and elongation stages for specific loci. This suggests that a subsequent oxidation of 5hmC into 5fC could happen, possibly followed by decarboxylation into 5caC as recently hypothesized [67].

## Conclusions

Numerous recent reports indicate the epigenetic topology of DNA vary both following normal differentiation processes and in response to developmental constraints. By combining restriction-based strategies, we provide here an important amount of genomic data illustrating two interesting points: first, the use of optimized RDA and HELP protocols in order to create two annotated maps identifying a total of 69,136 putative methylated and hydroxymethylated cytosines in the bovine genome during embryogenesis; secondly, the analysis of the obtained data in the context of bovine early embryonic development.

Thanks to a combined study of 5mC and 5hmC marks and the slower developmental kinetics of the bovine embryo compared to the mouse model, we were able to propose a complementary findings to those of Smith et al. [66] by showing that the unique regulatory phase of hypomethylation occurring during the blastocyst stage also lasts through differentiation (i.e. elongation-associated processes in the bovine embryo). Our data suggest that this hypomethylation status corresponds to a demethylation process with methylated cytosines at specific repeats (mostly Satellites and LTR) being converted to 5hmC during the blastocyst-elongation stage transition and likely to be targeted for complete demethylation.

The quality of our data is compatible with the design of an oligoset targeting these putative DNA methylation sensitive loci which is the basis for the ongoing development of a dedicated microarray-based platform for the purpose of systematic querying of the status of 5mC/5hmC marks during early development when submitted to environmental stresses.

## Methods

All reagents were obtained from Sigma-Aldrich (St-Louis, MO, USA) unless otherwise specified.

### Embryo *in vitro* production

Dairy cattle ovaries from a commercial slaughterhouse were transported to the laboratory in saline (0.9% NaCl) containing 1% antimycotic agent. Cumulus oocyte complex (COC) collection was conducted within 2 h of reception. COCs were aspirated from 2–6 mm follicles. Healthy COCs with at least five layers of cumulus were selected for maturation. Cumulus-oocyte complexes with fragmented cytoplasm, pyknotic cumulus, pale nuclei and abnormal morphology were rejected.

The COCs were washed in HEPES-buffered Tyrode’s medium (TLH) supplemented with 10% bovine serum, 200 μM pyruvate and 50 μg/mL of gentamycin. Groups of ten healthy COCs were placed in 50-μL droplets of medium under 9 mL of filtered mineral oil. Maturation medium was composed of TCM199 (Gibco 11150–059; Invitrogen, Burlington, ON, CAN), 10% foetal bovine serum (Sterile Fetal Bovine Serum for Cell Culture, Medicorp, Montréal, QC, CAN), 200 μm pyruvate, 50 μg/mL of gentamycin and 0.1 μg/mL of follicle stimulating hormone (FSH) (Gonal-f, Serono Canada Inc., Mississauga, QC, CAN). Droplets containing COCs were incubated for 24 h at 38.5°C with 5% CO_2_, 20% O_2_ and high humidity.

Matured COCs were washed twice in TLH. Groups of five matured COCs were added to 50-μL droplets of medium under filtered mineral oil. Each droplet consisted of modified Tyrode’s lactate medium, supplemented with 0.6% bovine serum albumin (Sigma fraction V), 40 mM pyruvate and 50 μg/mL gentamycin. A solution 1 mM hypotaurine, 2 mM penicillamine and 250 mM epinephrine was then added (2 μL) to the COC-containing droplets. The cryo-preserved pool of spermatozoa came from five Holstein bulls (Centre d’insémination artificielle du Québec). Semen was thawed in water at 37°C, laid on a discontinuous Percoll gradient (2 mL of 45% Percoll over 2 mL of 90% Percoll) and centrifuged at 700 × g for 30 min at room temperature. The pellets were re-suspended in *in vitro* fertilization medium to obtain a ratio of 50,000 spermatozoa/droplet. Fertilization took place in an incubator for 15–18 h at 38.5°C with 5% CO_2_, 20% O_2_ and high humidity.

Zygotes and unfertilized COCs were mechanically denuded by repeated pipeting and washed twice in TLH to ensure complete removal of cumulus cells. For standard culture conditions, groups of ten embryos were placed in 10-μL droplets of modified synthetic oviduct fluid (SOF) under filtered mineral oil [68]. Sequential SOF media were used as described previously [69]. The embryos were first placed in SOF#1 (6 mM lactate, 0.2 mM glucose). The embryo culture dishes were incubated 38.5°C with 6.5% CO_2_, 5% O_2_ and high humidity. Embryos were transferred to droplets of SOF#2 (1 mM lactate, 0.5 mM glucose) 72 h after fertilization and to droplets of SOF#3 (1 mM lactate, 2.5 mM glucose) 120 h after fertilization. On day 7 post-fertilization, blastocysts were collected, washed three times in nuclease-free phosphate-buffered saline (PBS) and frozen at −80°C until DNA extraction.

### Day-12 elongated embryo collection

Day-12 elongated embryos were produced at Alliance Boviteq inc. (Saint-Hyacinthe, QC, CAN). On days 8–12 post-oestrus, follicles with a diameter larger than 8 mm were aspirated. Thirty-six hours later, FSH (Folltropin-V, Bioniche Animal Health, Belleville, ON, CAN) was administered to fertile heifers. Overall, eight injections were given, twice daily, for a total of 400 mg of FSH in decreasing doses starting from 60 mg for the first dose to 20 mg for the last dose. Two doses (500 μg each) of prostaglandin F2α analogue (Estrumate, Intervet, Kirkland, QC, CAN) were administered with the two final FSH injections to trigger luteolysis. The animals exhibited oestrus about 36 h after the final FSH/Estrumate injection and were inseminated twice, 12 h and 24 h post-oestrus. Embryos were recovered by uterine flushing 12 days after the first insemination. Two elongated embryos of good quality were washed in nuclease-free (PBS) and frozen at −80°C until DNA extraction.

### Extraction of embryo genomic DNA

A total of 1,090 blastocysts (Day 7) produced *in vitro* in our lab and stored at −80°C were pooled prior to genomic DNA (gDNA) extraction in 500 μl of PBS. The same procedures were followed for Day 12 embryos. This volume was divided in two. Genomic DNA was then extracted from each 250-μl fraction using the “Micro protocol” from SwitchCharge extraction kit (Invitrogen) with a final elution in 150 μl of Tris–HCl (pH 8.5). The two resulting extractions were pooled. The gDNA quality was determined by electrophoretic migration and the gDNA was stored at −20°C. This blastocyst gDNA extraction was used to apply the three protocols targeting methylated sequences. Additional pools of 60 blastocysts and Day-12 embryos were extracted using the same protocol and used for q-PCR validation.

### Methylation-sensitive and Hydroxymethylation-sensitive digestion of gDNA

To enrich the methylated fraction in CG-rich regions, blastocyst and elongation-stage gDNAs were cleaved by MspI (C/CGG-specific) to form Tester DNA or by its methylcytosine-sensitive isoschizomer HpaII to form Driver DNA. Samples containing 350 ng of DNA were held for 12 h at 37°C with 10 U/μl MspI in 10× Buffer 4 or 20 U/μl HpaII in 10× Buffer 1 (New England Biolabs, Ipswich, MA, USA), adding the enzymes in three doses. DNA digests (100 ng) were analyzed by electrophoresis confirm cleavage. To enrich the hydroxymethylated fraction, cleavage was carried out as follows: 1) Tester – the DNA sample was cleaved with FspBI (C/TAG-specific, 10 U in 2× Tango Buffer, Fermentas Life Sciences, Lithuania) added in three doses, drawing into a pipette to mix. 2) Driver – the sample was cleaved with hydroxymethyl-sensitive isoschizomer BfaI (5 U in 10× NEB4, New England Biolabs) under the same conditions. DNA digests (100 ng) were analyzed by electrophoresis confirm cleavage. The remaining 250 ng were stored at −20°C.

### Adaptor ligation for HMe-RDA or Me-RDA

The double-stranded GC-overhang J adaptor (for Me-RDA) fits with MspI/HpaII ends, and the double-stranded TA-overhang JTA adaptor (for HMe-RDA) fits with BfaI ends. In order to use an enzyme cocktail to obtain only –TA protruding ends, we designed adaptors creating a new BfaI/FspBI site allowing one-step adaptor removal in all cases. Adaptor sequences are shown in Table 5. The double-stranded adaptors were obtained from Integrated DNA Technologies (Coralville, IA, USA) and diluted in TE buffer (pH 8) to 60 pmol/μl. Digested DNA previously purified using phenol-chloroform was dissolved in 10 μl of TE buffer. The ligation mixture with 10 μl of purified digested DNA (~150 ng) was mixed with 450 pmol of J duplex adaptor or JTA duplex adaptor, 10X T4 DNA ligation buffer (New England Biolabs) and water to obtain a total volume of 30 μl. The reaction was started at 55°C and slowly tempered until a temperature of 15-20°C was reached. T4 DNA ligase kept on ice was added (400 U) and the ligation reaction was performed in a thermal-cycler at 16°C for 16 h. The ligation products were diluted with 220 μl of TE buffer for subsequent amplification.

**Table 5:** Adaptors used for the preparation of methylation site libraries

**Name**	**Sequence**
J-1B	5′-ACCGACGTCGACTATCCATGAACC---3′
J-1A	3′---------------AGGTACTTGGGC-5′
JTA-1B	5′-AGTTACATCTGGTAAGTCTAG---3′
JTA-1A	3′-------TAGACCATTCAGATCAT-5′
N-1B	5′-AGGCAACTGTGCTATCCGAGGGAC---3′
N-1A	3′---------------AGGCTCCCTGGC-5′
NTA-1B	5′-ATCCTGAGTCTCTATATGGTCTAC---3′
NTA-1A	3′---------------ATACCAGATGAT-5′
AATT-1b	5′-AGTTACATCTGGTAGTCAGTCTC-----3′
AATT-1a	3′-------TAGACCATCAGTCAGAGTTAA-5′

### Genome representational PCR

In order to generate sufficient amplified product for subsequent subtraction steps, four representational PCR amplifications for Tester and 14 for Driver were performed using J-1A and JTA-1A primers. The reaction mixture contained TaKaRa Mg^2+^-free 10X reaction buffer (10 μl), 25-mM MgCl_2_ (6 μl), 2.5 mM dNTP (12.8 μl), 120 pmol of primer, *Ex Taq DNA polymerase* (10 U) plus 15 μl of diluted ligated DNA and water to a final volume of 100 μl. The 30 amplification cycles consisted of 1 min at 95°C, 1 min at 65°C and 1 min at 72°C for J-1B primer and 1 min at 95°C, 1 min at 65°C and 2 min at 72°C for JTA-1B primer. Tester and Driver amplicons were pooled and cleaned twice with phenol-chloroform for subsequent enzymatic cleavage of J and JTA adaptors. Ten μg of cleaved Tester amplicons were then loaded on 1.5% agarose gel, extracted and purified using the Qiagen gel extraction kit (Qiagen) in order to eliminate free J and JTA adaptors.

### Hybridization and subtraction for Me-RDA and HMe-RDA

Before the subtraction step, additional N or NTA adaptors were ligated to Testers only. The double-stranded GC-overhang N adaptor for Me-RDA and TA-overhang NTA adaptor for Me-RDA cocktail (Table 5) fit respectively MspI/HpaII and BfaI/FspBI ends. The ligation mixture with 10 μl of purified Tester DNA (~1 μg) was mixed with 450 pmol of N duplex adaptor or NTA duplex adaptor (60 pmol/μl), 10X T4 DNA ligation buffer and water (total volume of 30 μl). The ligation reaction was performed as described above. The ligation products were diluted with 50 μl of TE-solution containing 28 μg/ml tRNA for subsequent hybridization.

Tester (400 ng) and Driver DNA (40 μg) were co-purified with phenol-chloroform and precipitated using ammonium acetate and ethanol. The Tester-Driver co-precipitate was dissolved in 5 μl of EEB buffer (Sigma EPPS buffer 30 mM, pH 8 with 3 mM EDTA). After a 3-min denaturing step at 98°C, 1.5 μl of NaCl 5 M was added. Hybridization was carried out for 24 h at 67°C in a thermal cycler. The hybridization product was mixed with 40 μg of tRNA and diluted with 190 μl of Tris-EDTA. Subtractive amplification consisted of PCR for ten cycles as described above but using N-1A or NTA-1A primers, 10 μl of hybridization buffer, two tubes per hybridization, pooled, purified with phenol-chloroform and dissolved in 40 μl of TE, followed by digestion of half of this material for 30 min at 30°C with 20 U of mung bean nuclease (New England Biolabs) in order to remove single-stranded DNA (in 10X buffer plus water to 40 μl). Tris–HCl (50 mM, pH 8.9) was added and the mixture was held at 98°C for 5 min to inactivate the nuclease. This amplification was repeated on 10 μl of mung bean nuclease digest using the same N-1A and NTA-1A primers, but with a 20-cycle PCR.

### Digestion of gDNA and adaptor ligation for HELP cocktail

For targeting fragments preferentially outside of CpG sites, blastocyst (Day 7) and Day 12 gDNA were cleaved at 65°C with TasI enzyme (350 ng with 10 U in 2× Tango Buffer, Fermentas Life Sciences) added in three doses during the 12-h reaction. After testing by electrophoresis, the remaining 250 ng were stored at −20°C.

After TasI cleavage, the double-stranded adaptor resulting from annealing of AATT-1a and AATT-1b oligonucleotides (Schumacher, 2006; Table 5) was ligated to AATT-overhangs under the same conditions as for Me-RDA. Purified DNA (~150 ng in 10 μl) was mixed with 180 pmol of AATT-duplex adaptor (IDT), 10X T4 DNA ligation buffer (New England Biolabs) and water to 30 μl. The ligation product was diluted with 70 μl of water, mixed with 10 μg of tRNA, further purified with phenol-chloroform and precipitated in 20 μl of TE buffer for subsequent cleavage.

### Methylation-sensitive cleavage for HELP cocktail

For enrichment of the 5mC hyper-methylated fraction of the TasI cleavage product, a cocktail of three methylation-sensitive enzymes, HpaII (C/CGG), AciI (C/CGC) and HinP1I (GC/GC) was used. AATT-ligated DNA from blastocysts and Day 12 embryos, purified using phenol/chloroform/isoamylic alcohol 25:24:1, was cleaved (~70 ng in 14 μl) with 10 U/μl in 2X Tango Buffer, Fermentas Life Sciences) for 12 h at 37°C. The methyl-sensitive digestions were mixed with 10 μg of tRNA, further purified with phenol-chloroform and precipitated in 20 μl of TE buffer for subsequent PCR.

### HELP cocktail PCR

PCR amplification of the purified DNA digest (~50 ng) was also carried out with AATT-1b primer (120 pmol) using TaKaRa Mg^2+^-free 10X reaction buffer (10 μl), 25-mM MgCl_2_ (8 μl), 2.5-mM dNTP (12.8 μl) and *TaqEx* enzyme RR01AM (5 U), plus water to a final volume of 100 μl. The mixture was subjected to 25 cycles of 1 min at 95°C, 1 min at 56°C and 2 min at 72°C.

### q-PCR validation

Three additional pools of blastocysts and Day 12 embryos were collected and genomic DNA extracted as detailed above. Methylated site families in Me-RDA and HELP, and hydroxymethylated site families in HMe-RDA were selected following PCR quantification in methyl/hydroxymethyl insensitive *versus* sensitive cleavage. Reverse and forward primers were designed using IDT primerquest (http://www.eu.idtdna.com/Scitools/Applications/Primerquest/) and the EmbryoGENE Genome Browser (http://www.emb-bioinfo.fsaa.ulaval.ca/bioinfo/html/index.html) tools. Primer sequence, annealing and fluorescence acquisition temperatures, amplicon size and GeneBank accession numbers are shown in Additional file 1: Table S1. The reaction mixture contained LightCycler 480 SYBR Green I Master (Roche Diagnostics) and the threshold cycle was detected using a LightCycler 480 (Roche Diagnostics). The amplicon melting curve profile and DNA sequence was also determined. The threshold cycle of the same quantity (200 pg) of non-cleaved DNA with the respective sensitive enzyme was determined and ΔCt was calculated for analysis of the proportion of specific methylation or hydroxymethylation for each restriction site. Statistical validation was based on a simplified Wilcoxon two-group test to compare the pools of each condition [70].

### Sequencing strategy

The product of the final amplification steps was purified using the Qiagen PCR purification kit (Qiagen, Mississauga, ON, CAN) and quality was assessed using a 1-μl sample aliquot on an Agilent 7500 DNA chip on a Lab-on-a-Chip 2100 Bioanalyser apparatus (Agilent Technologies, Mississauga, ON, CAN). The remainder of the sample was used for pyro-sequencing using a Roche/454 Genome Sequencer FLX system. Sequencing results are made available at http://www.emb-bioinfo.fsaa.ulaval.ca/DeMontera2012/DiffMeth/Data/.

### Identification of putative methylated and hydroxymethylated restriction sites

SeqClean (http://www.sourceforge.net/projects/seqclean/) and the UniVec database [71] were used to remove adaptors and scan for contaminants. HELP sequences were then scanned for HpaII, AciI and HinP1I restriction sites and reads lacking any of these sites were excluded. All reads from each library were then clustered using the USEARCH tool [72] with a 97% identity threshold. The resulting consensus sequences were scanned for repeats using RepeatMasker [[73] <http://www.repeatmasker.org>] with build 20110920 of the RepBase database [74]. Consensus sequences were aligned to the UMD3.1 assembly of the bovine genome [75] using BLAT [76]. Alignments with less than 92% identity over 92% of the sequence length were discarded. When more than one such alignment existed for a consensus sequence, all alignments for this sequence were also excluded from further analysis. To determine the location of putative methylated or hydroxymethylated restriction sites, the remaining aligned sequences were extended *in silico* to the nearest restriction site of interest up to a maximum of 1,000 bp. This means the nearest HpaII, FspBI and TasI sites for Me-RDA, HMe-RDA and HELP Cocktail, respectively. As the sites at both extremities of Me-RDA and HMe-RDA fragments are potentially (hydroxy)methylated, the CpGs at both ends of a fragment were labeled as putatively methylated and hydroxymethylated, respectively. For HELP Cocktail, the CpGs within all HpaII, AciI and HinP1I restriction sites in-between the identified TasI sites were labeled as potentially methylated sites. The set of all sites thus identified served as the basis for further analysis. The source code of the analysis pipeline described in this section can be obtained at http://www.emb-bioinfo.fsaa.ulaval.ca/DeMontera2012/. The alignment of reads and the identified sites can be visualized within the bosTau6 assembly of the EmbryoGENE genome browser at http://www.emb-bioinfo.fsaa.ulaval.ca/.

### Distribution of candidate sites

Coverage graphs of the putative methylated and hydroxymethylated restrictions sites were generated using the hgGenome tool from the UCSC Genome Browser [77]. Cytobands within these graphs are approximations adapted from [38]. Using BEDTools [78], sites were further categorized depending on whether they occurred within inter-gene, exon, intron or promoter regions as defined by the Gnomon annotation of the UMD3.1 assembly of the bovine genome (http://www.ftp.cbcb.umd.edu/pub/data/assembly/Bos_taurus/). Finally, the exon, intron and promoter regions of each gene were grouped together so that all sites were associated with a unique gene or inter-gene region. Lists of library specific regions were then compared, and Venn diagrams were generated using the softwares of [79] and [80].

### Availability of supporting data

The data sets supporting the results of this article are available in the EmbryoGENE genome browser repository, http://www.emb-bioinfo.fsaa.ulaval.ca/DeMontera2012/.

## Abbreviations

Bp: Base pair; 5caC: 5-carboxycytosine; 5fC: 5-formylcytosine; HELP: Hpa tiny enrichment ligation mediated PCR; HMe-RDA: HydroxyMethylation sensitive representational difference analysis; 5hmC: 5-hydroxymethylcytosine; ICSI: Intra cytoplasmic sperm injection; IVF: *In Vitro* fertilization; Me-RDA: Methylation sensitive representational difference analysis; 5mC: 5-methylcytosine; PCR: Polymerase chain reaction; TSS: Transcription starting site.

## Competing interests

The authors declare they have no competing interest.

## Authors’ contributions

BdM conceived the combined 5mC 5hmC profiling strategy, performed Me-RDA, HMe-RDA and HELP Cocktail experiments, collaborated in sequence data analysis and drafted the manuscript. EF provided the bioinformatics strategy, performed functional mapping, coverage graphs and corresponding tables and drafted the manuscript. HASS and DG performed q-PCR validations and provided q-PCR methylation and hydroxymethylation profiles. DG contributed to the manuscript. IL produced *in vitro*-generated blastocyst embryos. PB provided *in vivo*-generated elongated embryos and contributed to the manuscript. MAS and CR conceived the general strategy to target embryo methylome. BdM, MAS and CR drafted the final manuscript. All authors read and approved the final manuscript.

## Supplementary Material

Additional file 1: Table S1 Describes PCR primer sequences and reaction conditions for methylation/hydroxymethylation validations. **Table S2.** Provides the list of the five most abundant repeated element within each library. **Table S3.** Presents an estimation of data quality (percentage of false positives) in our methods in comparison to others. **Figure S1.** Illustrates the genomic coverage of putative methylated/hydroxymethylated restriction sites on all bovine chromosomes. **Figure S2.** Shows the chromosomal distribution of BTSAT4 elements and of sites identified within the Me-RDA libraries. Click here for file
