# Supplementary material for: Combined methylation mapping of 5mC and 5hmC during early embryonic stages in bovine
Source: BMC Genomics. 2013 Jun 18;14:406. doi: 10.1186/1471-2164-14-406 (PMC3689598; doi:10.1186/1471-2164-14-406)
Supplement: Additional file 1: Table S1 — Describes PCR primer sequences and reaction conditions for methylation/hydroxymethylation validations. Table S2. Provides the list of the five most abundant repeated element within each library. Table S3. Presents an estimation of data quality (percentage of false positives) in our methods in comparison to others. Figure S1. Illustrates the genomic coverage of putative methylated/hydroxymethylated restriction sites on all bovine chromosomes. Figure S2. Shows the chromosomal distribution of BTSAT4 elements and of sites identified within the Me-RDA libraries. [file 1471-2164-14-406-S1.pdf]

Supplementary Table 1. PCR primer sequences and reaction conditions for methylation/hydroxymethylation validations.

5mC target number	Primer sequence 5' - 3'	Amplicon size (bp)	Annealing / Melting temp. (°C)	Genomic coordinates	Description
Me-RDA					
1	F: ACTCAAGAGCAGAGCAGGGTTT<br>R: TGTCCAGAAGCCAATGTTCC	122	54/82	3 loci	BTSAT4<br>including CHMP4B gene
2	F: GAACAGAGCGGGGTTCTTGG<br>R: GTGCCGGAAAACCTCGGTG	447	57/83	chr1:45,730,255-45,730,701	BTSAT4 in ABI3BP gene
3	F: TTCCTGATCTTCGCCTACG<br>R: TTGCTCTCGAGCCATGGTA	89	54/82	25 loci	BTSAT4<br>including ADAMTS3 gene
4	F: GGTGCCGGAAAACCTCAGTG<br>R: GAGCGGGGTTCTTTGCTTCA	442	57/84	7 loci	BTSAT4<br>including SLC9A10 gene
5	F: ACCGGGTTTCGGGAACTTTGT<br>R: AAGATGAAGCCCTTTCCCGCT	107	57/85	chr27:6,860,721-6,860,833	BTSAT4
6	F: TTCTCGAGTTACGGCGGGAT<br>R: AGAGCGGGCTTCTTGCTTCA	248	57/83	chr27:6,862,265-6,862,527	BTSAT4
7	F: ATTGCTCCAGAGCCATGGGA<br>R: AGGTCCTTCCCACCTCCAGTTT	177	57/83	chr13:7,751,833-7,752,009	BTSAT4 in MACROD2 intron
8	F: TTGCTCACGAGCCATGGTA<br>R: AAGACGAGGGAACACAGGGT	105	57/86	chr13:7,750,436-7,750,540	BTSAT4 in MACROD2 intron
9	F: TCGACCCTCCTGTCCAGAT<br>R: TCATCGCAAGATGAAGCCCT	206	57/85	1047 loci	BTSAT4
10	F: AACCTCAGTGTTCCTCTCG<br>R: AATTGGAGGTCGAAAGGGC	90	54/86	745 loci	BTSAT4
11	F: AAGACAATGTTCCTCTCCGGG<br>R: AAGAGATACCCGTCGCGATT	132	54/86	chr23:48,068,660-48,068,791	BTSAT4

Supplementary Table 1 (Part 2)

5hmC target number	Primer sequence 5' - 3'	Amplicon size (bp)	Annealing / Melting temp. (°C)	Genomic coordinates	Description
HMe-RDA					
1	F: AGCCGCCGCTCGAGTACA<br>R: ACGTGGTTTTTCTCGGGTTGT	448	57/83	chr19:61,927,256-61,927,703	BTSAT4 in ABCA5 gene
2	F: GCCCACAGAGCTCACTGAC<br>R: CAAGCTGGGGATTGCTCTCG	448	57/83	160 loci	BTSAT4
3	F: TGCTCCTTCCCGGGCG<br>R: AGGTCCCGGTGCCCTG	450	57/88	6 loci	BTSAT6<br>including RLG1gene
4	F: GATCCACGCCATCAAAGGCT<br>R: GTGGGCCTTAGAAAGCATCACT	446	55/72	chr11:107,310,060-107, 310,507	80% BovB (LINE)<br>20% BTLTR1 (LTR)
5	F: TGTGATCCACGCCATCAAAGG<br>R: TGGGCCTTAGAAAGCATCACT	448	57/80	chr11:107,310,061-107,310,509	80% BovB (LINE)<br>20% BTLTR1 (LTR)
6	F: GTGGGGTCACAAAGAGTCGG<br>R: GGGCAAGGCTGGCTAAATGT	442	57/80	chr14:42,051,387-42,051,831	ZFHX4 gene

Supplementary Table 2. List of the five most abundant repeated elements within each library.

Repeated element name	Count	Percentage of all repeats within library
Me-RDA - Day 7		
BTSAT4	25,253	82.4%
BTSAT6	1,486	4.9%
ERV2-1-LTR_BT	1,473	4.8%
BTLTR1	515	1.7%
BTSAT3	412	1.3%
HMe-RDA - Day 7		
BovB	9,176	36.3%
BTLTR1	4,413	17.5%
BTSAT4	3,400	13.5%
L1_BT	1,288	5.1%
ART2A	975	3.9%
HELP cocktail - Day 7		
ART2A	13,022	36.6%
BovB	4582	12.9%
BOV-A2	3029	8.5%
Bov-tA2	1869	5.3%
BTSAT3	1323	3.7%

Repeated element name	Count	Percentage of all repeats within library
Me-RDA - Day 12		
BTSAT4	18,957	87.1%
BTSAT6	1869	8.6%
BTSAT2	345	1.6%
BTSAT3	147	0.7%
ERV2-1-LTR_BT	76	0.3%
HMe-RDA - Day 12		
BTSAT4	3,889	31.7%
BTSAT6	2,146	17.5%
BovB	1,875	15.3%
BTLTR1	1,068	8.7%
BOV-A2	352	2.9%
HELP cocktail - Day 12		
ART2A	8,319	24.2%
Bov-tA2	3,079	8.9%
BOV-A2	2,632	7.6%
BovB	2,599	7.6%
Bov-tA1	1,623	4.7%

Supplementary Table 3. Estimation of data quality.

Method	Me-RDA (5mC)	HELP Cocktail (5mC)	HMe-RDA (5hmC)	GLIB (5hmC)	Anti-CMS (5hmC)	Anti-5hmC pAb (5hmC)
Reads with intact internal restriction site[a]	6,9%	7,1%	3,9%	N/A	N/A	N/A
Estimate of unmethylated cytosine[b,c]	1.7%	N/A	1%	<1%	<0.1%	<3%

[a] Percentage of clean reads (for Blastocyst and Elongation embryos in Table 1) containing an intact restriction site for a methyl-insensitive endonuclease more than 10 nucleotides from the ends, indicative of incomplete genomic cleavage. [b] corresponding to [a] divided by 4, as for Me-RDA and HMe-RDA, the probability for a read to have at least a methylated or hydroxymethylated cytosine at one end is ¾, and to have unmethylated cytosines at both ends (false positive) is ¼ (theoretical estimate for equiprobables situations). [c] Figures for GLIB, Anti-CMS and Anti-5hmC pAb methods are corresponding to the presence of unmethylated or methylated cytosines (false positives) as published by (Pastor, 2011) and (Huang, 2011).

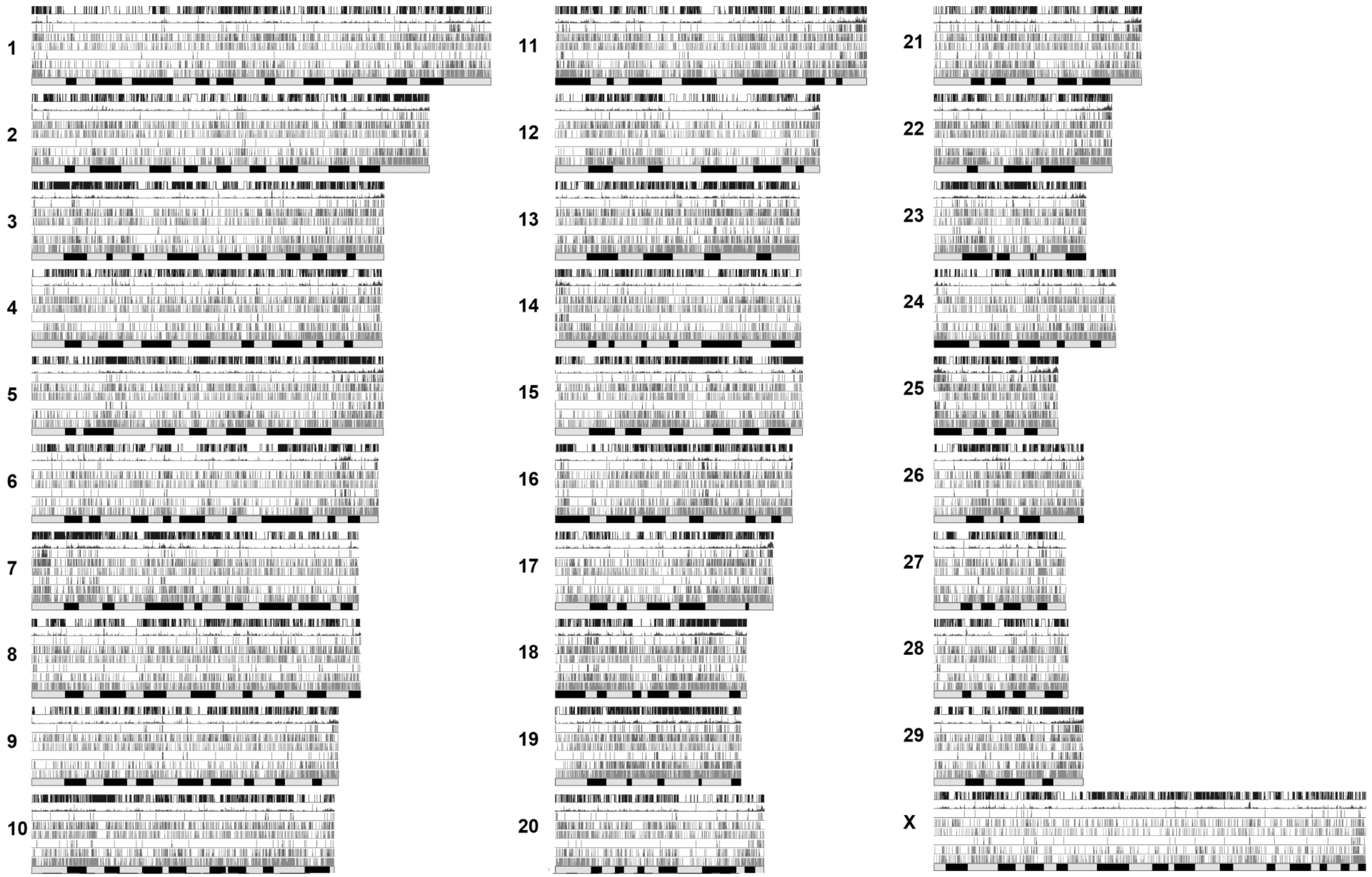

Supplementary Figure 1. Genomic coverage of putative methylated/hydroxymethylated restriction sites on all bovine chromosomes. Generated using the hgGenome tool of the UCSC Genome Browser. For each chromosome: chromatid representation with approximative cytobands (black and grey boxes) adapted from (Liao, 2007). For each chromosome, line represents: (1) gene coverage, (2) CpG islands depth, (3 -5) putative methylated or hydroxymethylated sites for Me-RDA, HMe-RDA and HELP cocktail libraries for Day 7 embryos, (6-8) the same for Day 12 embryos.

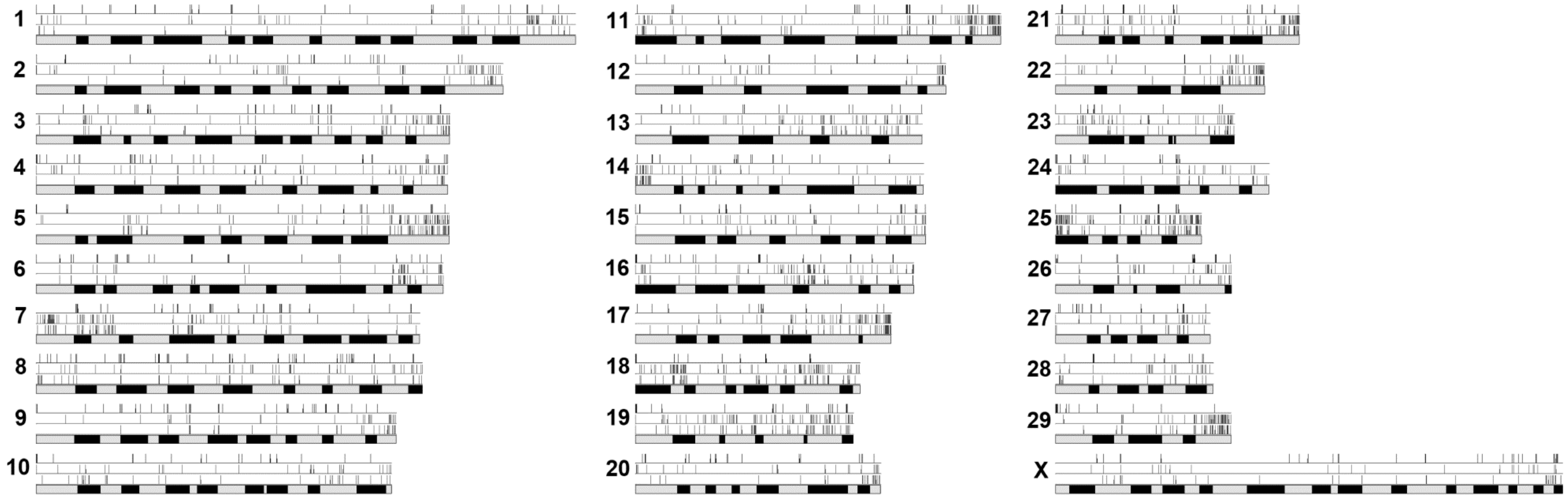

Supplementary Figure 2. Chromosomal distribution of BTSAT4 elements and of sites identified within the Me-RDA libraries. For each chromosome: approximative cytobands (black and grey boxes) adapted from {Liao, 2007}. Line 1: BTSAT4 elements. Line 2: sites identified in the Day 7 Me-RDA library. Line 3: sites identified in the Day 12 Me-RDA library.
